# Conversion of barley SNPs into PCR-based markers using dCAPS method

**DOI:** 10.1590/S1415-47572009005000047

**Published:** 2009-09-01

**Authors:** Fahimeh Shahinnia, Badraldin Ebrahim Sayed-Tabatabaei

**Affiliations:** Plant Genome Research Unit, National Institute of Agrobiological Sciences, TsukubaJapan; 2 Department of Agronomy and Plant Breeding, Isfahan University of Technology, IsfahanIran; 3 Department of Gene Bank, Leibniz Institute of Plant Genetics and Crop Plant Research, GaterslebenGermany; 4 Department of Biotechnology, Isfahan University of Technology, IsfahanIran

**Keywords:** *Barley (Hordeum vulgare L.)*, genome sequencing, mismatched primer, restriction enzyme, SNP genotyping

## Abstract

Molecular genetic research relies heavily on the ability to detect polymorphisms in DNA. Single nucleotide polymorphisms (SNPs) are the most frequent form of DNA variation in the genome. In combination with a PCR assay, the corresponding SNP can be analyzed as a derived cleaved amplified polymorphic sequence (dCAPS) marker. The dCAPS method exploits the well-known specificity of a restriction endonuclease for its recognition site and can be used to virtually detect any SNP. Here, we describe the use of the dCAPS method for detecting single-nucleotide changes by means of a barley EST, CK569932, PCR-based marker.

The progress of genome sequencing projects in model plants like barley, combined with the recent advances in high throughput assays, have provided a wealth of sequence information and SNP discovery. SNPs are genetic markers which are bi-allelic in nature, besides being highly abundant and less prone to mutations than SSRs (Kota *et al.*, 2003). They can contribute directly to a phenotype or can be associated with a phenotype as a result of linkage disequilibrium. In plants, SNPs are particularly useful in the construction of high resolution genetic maps, the positional cloning of target loci, marker assisted breeding of important genes, genome wide large-scale linkage disequilibrium associate analysis, DNA fingerprinting, and species origin, relationship and evolutionary studies.

Most conventional trait and molecular markers, such as restriction fragment length polymorphism (RFLP) and cleaved amplified polymorphic sequence (CAPS), are based on SNPs, *i.e.*, nucleotide substitutions or insertions/deletions (Nasu *et al.*, 2002). With the influx of various SNP genotyping assays in recent years, there has been a need for an assay that is not only robust, but also cost effective, simple and highly accurate. The available SNP genotyping methods can be classified into non-gel and gel based detection systems. Most of the non-gel based systems, such as SnaPshot, pyrosequencing and biplex invader, are based on known sequence information, and tend to require a relatively large initial automated investment (Pati *et al.*, 2004). In contrast, gel based methods are relatively low in start-up cost and moderate in throughput. In this context, CAPS markers are the most commonly used (Thiel *et al.*, 2003). The existence of nucleotide sequence polymorphism generation, a restriction site difference between varieties/lines to be analyzed, is essential for converting SNPs to CAPS markers. However, Michaels and Amasino (1998) and Neff *et al.* (1998) demonstrated that single-base changes generating no restriction site difference could be employed for the development of PCR-based markers by the derived CAPS (dCAPS) method. On using this method, a restriction enzyme recognition site which includes the SNP is introduced into the PCR product by a primer containing one or more mismatches to template DNA. The PCR product modified in this manner is then subjected to restriction enzyme digestion, and the presence or absence of the SNP is determined by the resulting restriction pattern. Like the CAPS technique, the dCAPS method is simple and relatively inexpensive (Neff *et al.*, 1998). The method has the potential to increase the number of molecular markers available for fine genetic mapping and the map-based cloning of genes.

The fertility of lateral spikelets in barley is controlled by the intermedium spike-c (*int-c*) gene, located on the telomeric region of the short arm of chromosome 4H (Komatsuda and Mano, 2002). Twenty six CAPS markers have been developed to enrich marker content around the gene (Shahinnia and Komatsuda, 2008). Here we are presenting the utilization of the dCAPS method to develop an additional PCR-based EST marker, CK569932, that could facilitate positional cloning of the gene in further studies. The advantages of the dCAPS method are also described.

Total DNA was extracted from the green leaves of Azumamugi (AZ) and Kanto Nakate Gold (KNG) barley cultivars (parental lines) and a derived 99 F_12_ recombinant inbred lines (RILs) population, as described by Komatsuda *et al.* (1998). The CK569932 expressed sequence tag (EST) generated from the barley BAC accession number AC087181 was selected from NCBI. The EST gave a significant match to rice chromosome 3 (*japonica* chromosome 3 psudomolecule, AP008209 full length sequence), orthologous with barley chromosome 4H, when using the Gramene database. PCR primers were designed with Oligo5 software (W. Rychlick, National Bioscience, Plymouth, MN, USA) and synthesized commercially (Bex, Tokyo, Japan). PCR amplifications were carried out in 10 μL reactions, each containing 0.25 U Ex*Taq* polymerase (Takara, Tokyo, Japan), 0.3 μM of each primer, 200 μM dNTP, 1.0 mM MgCl_2_, 1x PCR buffer 25 mM TAPS pH 9.3 (at 25 °C), 50 mM KCl, 1 mM 2-mercaptoethanol and 20 ng genomic DNA. The PCR programme consisted of a denaturation step of 94 °C/5 min, followed by 30 cycles of 94 °C/30 s, 58-62 °C/30 s and 72 °C/30 s, and a final incubation step of 72 °C/7 min.

PCR products were purified by using a QIAquick PCR purification kit (Qiagen, Germantown, MD, USA), and subjected to cycle sequencing by means of a Big Dye kit (Applied Biosystem, Foster, CA, USA). Sequencing reactions were purified on Sephadex G-50 (Amersham Pharmacia Biotech AB, Uppsala, Sweden) and analyzed with an ABI prism 3100 genetic analyzer (Applied Biosystem). Sequence information obtained from AZ and KNG were aligned by ClustalW software to identify nucleotide insertions/deletions (InDels). Since no restriction site(s) adjacent to the target SNP were identified, mismatched primers were designed by using the dCAPS Finder 2.0 program (Neff *et al.*, 2002). The PCR product obtained from a mismatched primer was digested with the restriction enzyme *Ssp*I (NIPPON GENE, Tokyo, Japan) by adding 10 μL of PCR products to 5 μL of a 10X buffer B containing 1.5 units of restriction enzyme. The samples were incubated for 3 h at the 37 °C temperature recommended by the manufacture. Following digestion, samples were separated by gel electrophoresis on 2.5% MetaPhor agarose (Cambrex Bio Science Rockland Inc., Rockland, MA, USA) in 0.5X TBE buffer and visualized through ethidium bromide staining.

The derived amplicon from nucleotide sequences of the CK569932 primer set (F: 5'-ACATTTCACAACCTC GTCAAG- 3' and R: 5'- GTGCACATTTCAAGCTAAG CC- 3') was successfully PCR amplified as a single product. Visualization by gel electrophoresis showed no polymorphic bands between parents. Hence the PCR product was subjected to cycle sequencing. Alignment of the sequence information revealed the SNP region as containing a purine (A)/pyramidine (T) point mutation between AZ and KNG parents. Since this SNP was not involved in the restriction sites of any enzyme, dCAPS primers were designed to create a suitable site. For this, we considered AZ as wild-type and KNG as mutant parents, and entered 25 nucleotides on each side of the SNP (point mutation) from haplotypes to the dCAPS Finder 2.0 program and allowed for one mismatch in the dCAPS primer.

As a result, 6 potential primer sequences for dCAPS analysis were displayed, including the high-lighted mismatches used to generate the restriction endonuclease recognition site (Figure 1). Based on the availability and cost of the restriction enzyme, 3 primer sequences corresponding to *Ssp*I, *Tsp*EI and *Mae*I were selected as forward primers, in addition to 2 reverse primers to form 6 combinations (Table 1). DNA fragments were successfully amplified, but the best amplification was obtained from dCAPS CK569932 (023-661) 058A080, this containing a mismatch (A), 5 nucleotides distant from the 3' end of the forward primer sequence and CK569932 (023-661) 329 as a reverse primer (Table 1). The PCR product of the primers was digested with restriction enzyme *Ssp*I. The enzyme cut the DNA fragment of the AZ parent at nucleotide 80 within the related template sequence. The molecular size of amplified fragments from AZ and KNG were 271 and 298, respectively, and MetaPhore 2.5% gel electrophoresis clearly separated polymorphic bands. The genotype of RILs and heterozygous (control) plants could rapidly discriminate when using this marker, due to the co-dominant nature of dCAPS (Figure 2). The CK569932 was located 18 cM distal from the telomeric region of the short arm of chromosome 4H in AZ KNG mapping population.

The conversion of SNP sites into CAPS markers by the artificial introduction of restriction sites involves the creation of mismatched primers whose successful application is not always trivial, depending on the number, positions and types of mismatches (Thiel *et al.*, 2003). Generally, dCAPS primers that failed either in PCR reproducibility or restriction enzyme digestion are those having more than one mismatch close to the 3' end of the primer sequence (Umali and Nakamura, 2003). In addition, the strict nature of the mismatch could also affect the efficacy of the primer, since purine-purine mismatches are less stable than pyramidine-pyramidine. However, the success of using dCAPS markers for detecting single nucleotide polymorphism has been reported in wheat, rice, bananas and *Arabidopsis*, as well as barley (Iwaki *et al.*, 2002; Yanagisawa *et al.*, 2003; Komori and Nitta, 2005; Umali and Nakamura, 2003; Neff *et al.*, 1998). It is worthy to mention that the cost of restriction endonuclease used in assays is also one of the more important criteria for primer designing (Neff *et al.*, 2002).

Many of the new SNP assays rely on detection methods that require highly sophisticated instrumentation. These specialized instruments are very potent but come at prices which are prohibitive for most laboratories. Moreover, many assays require expensive probes or other reagents that escalate genotyping costs beyond that which is affordable. dCAPS markers are primarily used when the SNP of interest does not alter the restriction site of an available restriction enzyme. The method can also be used to introduce a specific restriction site for each of the two alleles being analyzed, in order to positively identify the homozygote for a particular allele without the possibility of miss-scoring due to partial restriction enzyme digestion. In some cases, it is also possible to design dCAPS primers that create a suitable site adjacent to the less costly enzyme. This would be useful when a large number of plants is to be genotyped for a CAPS marker. The other situation where dCAPS markers are useful is when a CAPS assay for detecting a particular SNP is not usable due to the presence of an additional restriction site very close to the CAPS polymorphism to be analyzed. In this situation, a dCAPS marker can be generated that uses a primer-induced mismatch to disrupt the nearby second restriction site (Neff *et al.*, 2002).

**Figure 1:**
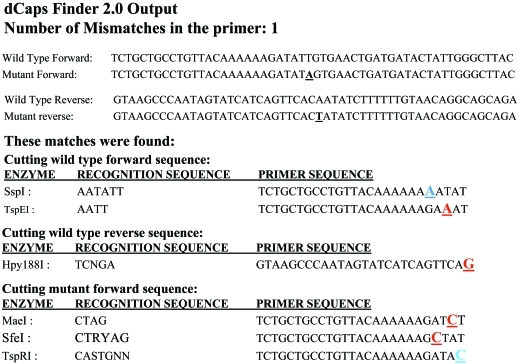
Output of SNP analysis for AZ (wild-type) and KNG (mutant) parents. (A) The point mutation is a conversion of A to T (bold underlined) nucleotide. The primer is allowed one mismatch. All sequences are written from 5' to 3'. (B) Mismatched nucleotides are underlined. Red, blue and green letters represent purine-purine, purine-pyrimidine and pyrimidine-pyrimidine mismatches, respectively.

**Figure 2:**
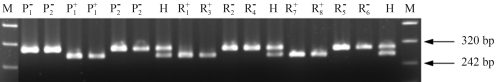
Electrophoresis profiles of dCAPS product. P_1_: AZ, P_2_: KNG, +: cleaved type by *Ssp*I enzyme, **-**: uncleaved type, H: hetero type (control), R: RIL and M: DNA molecular weight marker 8. Molecular size of DNA fragments are 271 bp (P_1_^+^) and 298 bp (P_2_^-^).

When referring to many factors for SNP genotyping, the size of amplified PCR fragments is critical. Earlier studies have shown that many assays, such as single strand confirmation polymorphism (SSCP) and pyrosequencing, are extremely sensitive and often fail to work in the case of amplicons above 200 and less than 500 bp, respectively (Nataraj *et al.*, 1999; Ahmadian *et al.* 2000). Among these SNP genotyping assays, only the dCAPS method would not be limited by amplicon size. In conclusion, the method is rapid and does not require the large quantities of DNA, hybridization, radioactivity or optimization that other methods designed to detect point mutation employ.

## Figures and Tables

**Table 1:** Sequence, mismatch position, recognition site, restriction enzyme and expected product size of CK569932 dCAPS primers.

dCAPS primer	Primer sequence (5' to 3') ^a^	Mismatch position^b^	Recognition site	Restriction enzyme	Expected product (bp)^c^
(023-661)058A080	TCTGCTGCTCTGTTACAAAAAA*A*ATAT	5	AATATT	*Ssp*I	271/27
(023-661)058A082	TCTGCTGCTCTGTTACAAAAAAGA*A*AT	3	AATT	*TspE*I	269/29
(023-661)058C083	TCTGCTGCTCTGTTACAAAAAAGAT*C*T	2	CTAG	*Mae*I	268/30

^a^Italicized bold letters are mismatched base. ^b^Number corresponds to position of mismatched bases relative to the 3' end of forward dCAPS primers. Reverse primers are: (023-661)194: 5'- TCCAGCATGTAGAGTTATATTGAGCAG- 3' and (023-661)329: 5'- TCGATGCCTACAACAAATGGACCACCG- 3'. ^c^Expected product size of dCAPS primers after digestion.

## References

[Ahmadianetal2000] Ahmadian A., Gharizadeh B., Gustafsson A.C., Sterky F., Nyren P., Uhlen M., Lundeberg J. (2000). Single-nucleotide polymorphism analysis by pyrosequencing. Anal Biochem.

[Iwakietal2002] Iwaki K., Nishida J., Yanagisawa T., Yoshida H., Kato K. (2002). Genetic analysis of *Vrn-B1* for vernalization requirement by using linked dCAPS markers in bread wheat (*Triticum aestivum* L. ). Theor Appl Genet.

[KomatsudaandMano2002] Komatsuda T., Mano Y. (2002). Molecular mapping of the intermedium spike-c (*int-c*) and non-brittle rachis 1 (*btr1*) loci in barley (*Hordeum vulgare* L. ). Theor Appl Genet.

[Komatsudaetal1998] Komatsuda T., Nakamura I., Takaiwa F., Oka S. (1998). Development of STS markers closely linked to the *vrs1* locus in barley, *Hordeum vulgare*. Genome.

[KomoriandNitta2005] Komori T., Nitta N. (2005). Utilization of the CAPS/dCAPS method to convert rice SNPs into PCR-based markers. Breed Sci.

[Kotaetal2003] Kota R., Rudd S., Facius A., Kolesov G., Thiel T., Zhang H., Stein N., Mayer K., Graner A. (2003). Snipping polymorphisms from large EST collections in barley (*Hordeum vulgare* L. ). Mol Genet Genom.

[MichaelsandAmasino1998] Michaels S.D., Amasino R.M. (1998). A robust method for detecting single-nucleotide changes as polymorphic markers by PCR. Plant J.

[Nasuetal2002] Nasu S., Suzuki J., Ohta R., Hasegawa K., Yui R., Kitazawa N., Monna L., Minobe Y. (2002). Search for and analysis of single nucleotide polymorphisms (SNPs) in Rice (*Oryza sativa*, *Oryza rufipogon*) and establishment of SNP markers. DNA Res.

[Natarajetal1999] Nataraj A.J., Olivos-Glander I., Kusukawa N., Highsmith W.E.J. (1999). Single-strand conformation polymorphism and heteroduplex analysis for gel-based mutation detection. Electrophoresis.

[Neffetal1998] Neff M.M., Neff J.D., Chory J., Pepper A.E. (1998). dCAPS, a simple technique for the genetic analysis of single nucleotide polymorphisms: Experimental applications in *Arabidopsis thaliana* genetics. Plant J.

[Neffetal2002] Neff M.M., Turkand E., Kalishman M. (2002). Web-based primer design for single nucleotide polymorphism analysis. Trends Genet.

[Patietal2004] Pati N., Schowinsky V., Kokanovic O., Magnuson V., Ghosh S. (2004). A comparison between SNaPshot, pyrosequencing, and biplex invader SNP genotyping methods: Accuracy, cost, and throughput. J. Biochem Biophys Meth.

[ShahinniaandKomatsuda2008] Shahinnia F., Komatsuda T. (2008). Comparative mapping approaches for marker enrichment of barley chromosome 4H harboring intermedium spike-c (*int-c*) gene. Proceedings of Plant and Animal Genome XVI Conference.

[Thieletal2003] Thiel T., Michalek W., Varshney R.K., Graner A. (2003). Exploiting EST databases for the development and characterization of gene-derived SSR-markers in barley (*Hordeum vulgare* L. ). Theor Appl Genet.

[UmaliandNakamura2003] Umali R.P., Nakamura I. (2003). Identification of dCAPS markers that discriminate A and B cytoplasms in bananas (*Musa spp*. ). Plant Biotech.

[Yanagisawaetal2003] Yanagisawa T., Kiribuchi-Otobe C., Hirano H., Suzuki Y., Fujita M. (2003). Detection of single nucleotide polymorphism (SNP) controlling the waxy character in wheat by using a derived cleaved amplified polymorphic sequence (dCAPS) marker. Theor Appl Genet.

